# Identification of three novel mutations by studying the molecular genetics of Maple Syrup Urine Disease (MSUD) in the Lebanese population

**DOI:** 10.1016/j.ymgmr.2014.03.005

**Published:** 2014-07-12

**Authors:** Omar Tabbouche, Amer Saker, Harry Mountain

**Affiliations:** a Staffordshire University, New Mazloum Hospital, Tripoli, Lebanon; b Analytical Testing Laboratories, Beirut, Lebanon; c Staffordshire University, Molecular Biology & Genetics, Stoke on Trent, UK

**Keywords:** Maple Syrup Urine Disease, MSUD, Mutation, Lebanon, Lebanese population, Lebanon MSUD, MSUD novel mutations

## Abstract

Maple Syrup Urine Disease (MSUD) is a genetically heterogeneous metabolic disorder that is transmitted in an autosomal recessive manner. According to clinical data, MSUD prevalence in Lebanon is expected to be higher than the International prevalence because of consanguineous marriage. Novel mutations are still getting detected by using DNA sequencing for mutation analysis in MSUD patients.

In the current study, we have extracted DNA from Lebanese MSUD patients in order to amplify the exonic and flanking intronic regions of the genes implicated in MSUD (*BCKDHA*, *BCKDHB*, and *DBT*) and sequenced the resultant amplified products to assess the molecular genetics of MSUD in the Lebanese population studied.

All of the mutations identified occurred in the homozygous state, which reflects the high rate of consanguineous marriage in Lebanon. In the current study, we have identified one previously cited mutation and three novel mutations not previously described in the scientific literature. The identified mutations were distributed as follows: three patients (60%) had two nucleotide substitutions in the DBT gene (c.224G>A and c.1430T>G), one patient (20%) had a gross deletion in the BCKDHA gene (c.488_1167+3del), and one patient (20%) had a small deletion in the BCKDHB gene (c.92_102del).

The majority of the mutations identified in the Lebanese MSUD patients occurred in the DBT gene. Consanguineous marriage is a major risk factor for the prevalence of MSUD in Lebanon.

## 1 Introduction

Discovered by [9], Maple Syrup Urine Disease was first described as a fatal neurodegenerative disease characterized by a progressive infantile cerebral dysfunction and an unusual urinary substance, that lead to death by 3 months of age [9]. Three years after, this syndrome was referred to “Maple Syrup Urine Disease” by Westall et al., who has detected high levels of branched-chain amino acids (BCAAs) in the urine of the patients [18]. Fifty-eight years after its discovery, MSUD is now defined as an inborn error of branched-chain amino-acid metabolism caused by deficiency of a multi-enzyme complex, the “Branched Chain Keto-acid Dehydrogenase” (BCKD) [5]. This mitochondrial enzyme complex catalyzes the oxidative decarboxylation of branched-chain α-keto acids (BCKA) derived from transamination of the three branched-chain amino acids (BCAA) namely leucine, isoleucine, and valine [10].

MSUD is a biochemical genetic disorder that is inherited in an autosomal recessive manner. It is characterized by a maple syrup odor of urine and cerumen 12–24 h after birth, high levels of plasma BCAAs, ketonuria, irritability, poor feeding by 2–3 days of age, lethargy, deepening encephalopathy, intermittent apnea that aggravates to reach central respiratory failure, coma, and death in the first weeks of life if left untreated [6].

MSUD has a worldwide prevalence of 1/180,000 newborns [2]. In his study on the white population of New England, Levin (1993) found a frequency of MSUD of 1 in 290,000 according to statistics conducted on newborn screening [8]. However, the prevalence rate of MSUD tends to increase to 1/50,000 newborns in certain populations with a high rate of consanguineous marriages, and may even reach up to 1/176 in the Mennonite population residing in Pennsylvania, USA [3]. It is the most frequently occurring organic acidemia in Turkey [4]. According to the Israeli National Genetics Database (INGD), the estimated incidence of MSUD among non-Jewish living in Israel is about 1/40,000 live births [7].

DNA sequencing is the most sensitive molecular technique for the diagnosis and confirmation of MSUD. It comprises the sequencing of the exonic and flanking intronic regions of the three MSUD-related gene offers a 95% mutation detection rate, making it the most sensitive technique recommended by the US-CLIA [16].

The distribution of the mutations occurring in each of the three genes among MSUD patients varies widely between different populations. The mutational spectrum of MSUD has been assessed and identified in many countries (USA, Portugal, Italy, Germany, France, Turkey, Cyprus, China, India, United Arab Emirates, and Japan) and in different populations and communities (Mennonite, Gypsy Portuguese, and Ashkenazi).

To date, there are no studies performed on Lebanese MSUD patients so far. We do not even have an incidence rate of MSUD in Lebanon. Our aims are to identify and assess the genetic mutations of Lebanese MSUD patients, and to predict the effects of these mutations on the resultant proteins.

This study will be the first study to identify the molecular genetics of MSUD in the Lebanese population, which is of great interest to us because the Lebanese population is a mixture of various populations from different ethnic origins.

## 2 Materials & methods

### 2.1 Sample population

**Fig. 1:**
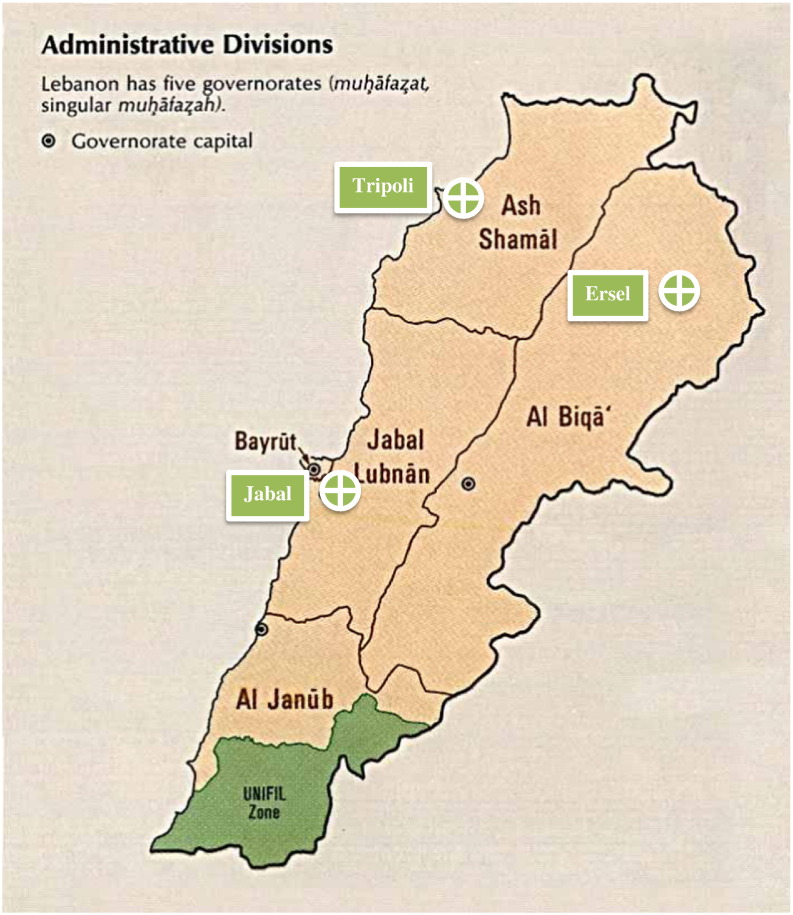
Geographical distribution of the identified mutations.

The frequency of MSUD worldwide is 1/180,000 newborns. The Lebanese population includes approximately 4,200,000 people [17]. By projection to the Lebanese population, the estimated number of MSUD patients in Lebanon would be 23 patients (Target population). Our sample population included 5 Lebanese MSUD patients (21.73% of target population), previously diagnosed with MSUD by Gas Chromatography Tandem Mass Spectrometry GC/MS/MS [11]. Sampling was achieved by using the convenience (non-probability) sampling method. For a detailed geographical distribution of our sample population, please refer to Fig. 1.

At the time of enrolment, an informed consent form explaining the aims and advantages of the study was delivered to the parents of each patient, read and signed upon their agreement.

Our study has got the Institutional Review Board approval from the New Mazloum Hospital, Tripoli, Lebanon.

A brief information questionnaire about the patient's information, family tree, ethnic origin, and MSUD phenotype was filled for each subject.

### 2.2 Peripheral blood samples

10 ml of peripheral blood was withdrawn from each of the enrolled patients, collected in EDTA tubes, and kept in the fridge (2–4 °C) for a maximum of 2 days prior to DNA extraction.

### 2.3 DNA extraction

Genomic DNA was extracted with the High Pure PCR Template Preparation Kit (Roche Diagnostics GmBH, Germany) according to the manufacturer's instructions.

### 2.4 Polymerase chain reaction

Our PCR and DNA sequencing protocols are taken from a previously published article [6].

A negative control (No DNA template) and a positive control (DNA template) were run in each PCR reaction to check for contamination and absence of amplification respectively.

PCR products were collected for detection of the mutations present by DNA sequencing.

### 2.5 DNA sequencing

The amplified products were analyzed by capillary sequencing using the ABI Genetic Analyzer 3130.

All primers are available upon request to the corresponding author by e-mail.

The resulting sequences were compared to the corresponding sequences in Nucleotide (BCKDHA: NM_000709.2, BCKDHB: NM_183050.2, DBT: NM_001918.2).

## 3 Results

In four out of the total of five patients recruited in our study, we had identified novel mutations not previously described in the scientific literature. One novel mutation occurred in two patients, while the two other novel mutations occurred in two patients.

Three patients showed mutations in the DBT gene, one had a mutation in the BCKDHA gene, and one had a mutation in the BCKDHB gene. So, the majority of mutations occurred in the DBT gene, while the minority was identified in the BCKDHA and BCKDHB genes. To extrapolate this data as percentages, 60% (3 patients) of the mutations were found in the DBT gene (MSUD Type II), 20% (1 patient) of the mutations were in the BCKDHA gene (MSUD Type IA), and 20% (1 patient) of the mutations occurred in the BCKDHB gene (MSUD Type IB).

Each patient has been given a reference number following the order cited in the sample population above.

As far as our study suggests, there is a distribution of mutations in all of the genes implicated in MSUD. However, the largest percentage corresponds to the DBT gene.

All mutations occur in the homozygous state. Each geographic area shares mutations in the same gene.

All of our patients were Arab-Muslims, however, they originated from different Muslim Doctrines. Also, patients 1 and 2 share the same doctrine (Shiite), while patients 3 and 4 share also another doctrine (Druze). However, patient 5 belonged to a third Muslim doctrine (Sunnit).

The first novel mutation identified is in the BCKDHA gene corresponding to MSUD type IA, it is a gross deletion starting at exon 5 and terminating at exon 8 (c.488_1167+3del).

The second novel mutation identified is a single nucleotide substitution occurring on the exon 3 (c.224G>A), where guanine at position 224 was substituted with Adenine. This resulted in an amino acid replacement from Glycine to Glutamic Acid at position 75 (p.Gly75Glu) in the E2 protein.

The third novel mutation was also identified in the DBT gene. It is also a single nucleotide substitution (c.1430T>G) that occurred at position 1430 in the cDNA corresponding at exon 11. This mutation resulted in an amino acid substitution in the resultant protein from Methionine to Arginine at position 477.

To summarize, mutations identified were of various types and had various outcomes on the resultant protein function. Alamut® software (Interactive Biosoftware, Rouen, France) was used to predict the effect of the mutation on the protein function.

## 4 Discussion

As we can conclude from our results above, all of our patients are homozygous for the mutations identified. Each patient has two identical alleles, one from the mother and one from the father. This clearly reveals the effect of consanguinity between the parents behind the occurrence of MSUD in Lebanon. Indeed, all of our patient's parents showed a degree of relativity except for one patient from Ersel. Since consanguinity is very common among the Lebanese population, we should expect to get a much higher incidence rate of MSUD in Lebanon than the international rate, which is 1/180,000 [1]. However, this can be proved in the future by a study on the rate of incidence of MSUD in Lebanon.

As for the geographical mutation distribution, the BCKDHA and BCKDHB deletion mutations occurred in the Jabal area above the capital of Lebanon, Beirut. The DBT mutations occurred in the Ersel area, North East of Lebanon, above the Bekaa valley, and in Tripoli, North Lebanon. However, the same mutation was identified in the two patients from Ersel, while the third patient from Tripoli had another mutation in the DBT gene.

### 4.1 Mutation types

In our study, we have identified four mutations in five MSUD patients (Table 1). Two mutations were a single nucleotide substitution, and the other two mutations were deletions, a small deletion and a gross deletion. Deletions occurred in the BCKDHA & BCKDHB genes, while nucleotide substitutions occurred also exclusively in the DBT gene. Since we had identified an identical mutation in two MSUD patients, we will take into consideration the number of patients rather than the number of mutations. By extrapolating our results as a percentage, 60% of the mutations identified were single nucleotide polymorphisms, while 40% were deletions.

#### 4.1.1 BCKDHA mutation (NP_000700.1, CCDS12581.1) [13]

The only BCKDHA mutation identified in our study is a gross deletion (c.488_1167+3del), that starts at exon 5 and terminates at exon 8 of the gene. We predict the deletion to be located in intron 4 rather than exon 5, which has led to the complete deletion of the exon 5 to exon 8 sequences. However, we could not identify the location because, technically, we cannot amplify and sequence intron 4 because of its very large size.

According to the Alamut® software for mutation interpretation, this gross deletion creates a frame shift starting at codon Val163 in the mature peptide, just after the Thiamine pyrophosphate-binding domain. The new reading frame ends in a Stop Codon 5 positions downstream. As an end result, this mutation has led to the complete loss of function of the E1A protein.

#### 4.1.2 BCKDHB mutation (NP_898871.1, CCDS4994.1) [14]

The only BCKDHB mutation identified is a 11 bp small deletion (c.del92_102) which occurs in exon 1 of the gene. It was previously identified and described by Nobukuni et al. [12]. It is localized in the region that targets the mitochondrial leader peptide of the E1B subunit. This small deletion (c.92_102del) has resulted in a substitution from Alanine to Phenylalanine at position 32 in the resultant protein, and a shift in the reading frame with appearance of premature Stop Codon 48 amino acids before (p.Ala32PhefsX48). This mutation is present in the transit peptide (1–50) amino acid sequence, and ends at position 48 just before the start of the mature peptide sequence (66–390). This mutation has led to the complete loss of function of the E1B protein, and a rapid degradation of the E1A subunit because of failure of assembly to the E1B subunit to form a stable E1 tetramer. Nobukuni has concluded that this deletion is probably caused by a “slipped mispairing” or “unequal chromosome crossing-over” [12].

#### 4.1.3 DBT mutations (NP_001909.3, CCDS767.1) [15]

The first mutation identified in the DBT gene was found in two patients from the same geographical area, although these patients were not related. This means that they share common ancestry. As described before, it is a single nucleotide substitution occurring on the exon 3 (c.224G>A). This mutation is present in the mature peptide sequence (62–482), exactly in the lipoyl-binding domain that extends from position 65 to position 138. So, the lipoyl Co-factor cannot bind to the mutated protein. As a result, we should expect a defect in the lipoyl-binding function of the E2 protein. According to Alamut® software, it is a disease-causing (p-value: 1.0), missense mutation giving rise to variations in the resultant protein domains “Biotin/lipoyl attachment” and “Single hybrid motif”. The resultant protein has moderate physicochemical difference from the original protein because of amino acid substitution. The net result is a complete deletion of function of the E2 protein. The resultant structure has 94% of residues modeled at > 90% confidence.

The second DBT mutation is also a single nucleotide substitution (c.1430T>G) that occurred at position 1430 in the cDNA corresponding to exon 11. This mutation resulted in an amino acid substitution in the resultant protein from Methionine to Arginine at position 477 (p.Met477Arg). This mutation occurs also on the mature peptide sequence (62–482). It is present in the catalytic domain of the E2 protein which extends from position 267 to position 479. As a result, we should expect a defect in the catalytic function of the E2 protein. According to Alamut® software, it is also a missense mutation giving rise to highly conserved nucleotide and amino acid sequences, and a moderate physicochemical difference in the resultant protein. This variation exists in the catalytic domain. It is a disease-causing mutation (p-value: 1.0), which results in a complete loss of function of the resultant protein. The resultant structure has 83% of residues modeled at > 90% confidence. The location of the mutation still produces an alpha helix starting at position 473 and terminating at position 479.

### 4.2 Genotype to phenotype correlation

The majority of our patients (3 patients) had the Intermediate MSUD phenotype, while the minority (2 patients) had the Classic MSUD phenotype. The Intermediate MSUD phenotype was linked to mutations exclusively identified in the DBT gene. The Classic MSUD phenotype was linked to deletions occurring in the BCKDHA and BCKDHB genes. As previously described, mutations in either the BCKDHA or BCKDHB genes alone will affect the folding and assembly of the mutated subunit protein with the other, which will affect the function of the resultant E1 heterotetramer even though mutations would have occurred in only one subunit only. The net result would be deletion of function of both subunits (E1A & E1B). This is not the case for mutations in the DBT gene, because the mutated E2 core would have lost its function, but the E1 heterotetramer is still intact.

There is still a lot of research work that has to be conducted on the Lebanese MSUD population. First of all, an accurate prevalence rate must be determined and published. Allele frequency of the Lebanese population and comparison with the Lebanese MSUD population are other research hypotheses that can be answered by a case–control study. As we can conclude, there are plenty of research questions in the topic of MSUD in the Lebanese population that need to be answered by future studies.

## Figures and Tables

**Table 1:** Characteristics of the identified mutations.

	Gene	Exon	Type	Mutation	Codon	Result	Phenotype	Reference
1	DBT	3	Substitution	c.224G>A	75	p.Gly75Glu	Classic	Novel
2	DBT	3	Substitution	c.224G>A	75	p.Gly75Glu	Classic	Novel
3	BCKDHB	1	Deletion	c.92_102del	31	p.Arg31Glnfs*16	Intermediate	[12]
4	BCKDHA	5–8	Deletion	c.488_1167+3del	163	p.Val163Glyfs*6	Intermediate	Novel
5	DBT	11	Substitution	c.1430T>G	477	p.Met477Arg	Intermediate	Novel
